# Had1 Is Required for Cell Wall Integrity and Fungal Virulence in *Cryptococcus neoformans*

**DOI:** 10.1534/g3.117.300444

**Published:** 2017-12-12

**Authors:** Won-Hee Jung, Ye-Eun Son, Sang-Hun Oh, Ci Fu, Hye Shin Kim, Jin-Hwan Kwak, Maria E. Cardenas, Joseph Heitman, Hee-Soo Park

**Affiliations:** * School of Food Science and Biotechnology, Institute of Agricultural Science and Technology, Kyungpook National University, Daegu 41566, Republic of Korea; † School of Life Science, Handong Global University, Pohang 37554, Republic of Korea; ‡ Department of Molecular Genetics and Microbiology, Duke University Medical Center, Durham, North Carolina 27710

**Keywords:** *Cryptococcus neoformans*, calcineurin, Crz1, fungal virulence, Had1

## Abstract

Calcineurin modulates environmental stress survival and virulence of the human fungal pathogen *Cryptococcus neoformans*. Previously, we identified 44 putative calcineurin substrates, and proposed that the calcineurin pathway is branched to regulate targets including Crz1, Pbp1, and Puf4 in *C. neoformans*. In this study, we characterized Had1, which is one of the putative calcineurin substrates belonging to the ubiquitously conserved haloacid dehalogenase β-phosphoglucomutase protein superfamily. Growth of the *had1*∆ mutant was found to be compromised at 38° or higher. In addition, the *had1*∆ mutant exhibited increased sensitivity to cell wall perturbing agents, including Congo Red and Calcofluor White, and to an endoplasmic reticulum stress inducer dithiothreitol. Virulence studies revealed that the *had1* mutation results in attenuated virulence compared to the wild-type strain in a murine inhalation infection model. Genetic epistasis analysis revealed that Had1 and the zinc finger transcription factor Crz1 play roles in parallel pathways that orchestrate stress survival and fungal virulence. Overall, our results demonstrate that Had1 is a key regulator of thermotolerance, cell wall integrity, and virulence of *C. neoformans*.

Calcineurin is a highly conserved serine/threonine protein phosphatase, which is activated by Ca^2+^/calmodulin in eukaryotic organisms, and plays diverse roles in controlling gene expression and cellular processes (Stewart *et al.* 1982; Rusnak and Mertz 2000; Aramburu *et al.* 2004). Increased intracellular Ca^2+^ levels in response to internal or external cues bind to calmodulin, and, in turn, the Ca^2+^-calmodulin complex interacts with the calcineurin catalytic subunit and activates the phosphatase (Crabtree 2001). Activated calcineurin dephosphorylates target proteins that then orchestrate the response and adaptation to stress (Kissinger *et al.* 1995; Aramburu *et al.* 2000). A key substrate of calcineurin is NFAT (nuclear factor of activated T cell), a transcription factor that regulates the expression of genes associated with T cell activation and the development of the nervous and cardiac systems (Northrop *et al.* 1994; Feske *et al.* 2003; Hogan *et al.* 2003; Aramburu *et al.* 2004). The calcineurin signaling pathway is inhibited by the immunosuppressive and antifungal compounds FK506 and cyclosporin A (CsA), which bind to FK506-binding protein 12 (FKBP12) and cyclophilin A, respectively, forming stable complexes. The FK506-FKBP12 and CsA-cyclophilin A complexes interact with calcineurin, thereby inhibiting phosphatase activity (O’Keefe *et al.* 1992; Schreiber and Crabtree 1992; Siekierka and Sigal 1992; Sigal and Dumont 1992; Kissinger *et al.* 1995).

While calcineurin is highly conserved in eukaryotes, the functions and biological roles regulated by calcineurin are distinct in mammals and fungi (Steinbach *et al.* 2007b). In the model yeast *Saccharomyces cerevisiae*, calcineurin is essential for stress responses, bud emergence, and cell cycle regulation (Miyakawa and Mizunuma 2007). In yeast, Crz1 (calcineurin responsive zinc finger 1) is a prominent calcineurin substrate that transcriptionally regulates mRNA expression of target genes associated with cell-wall synthesis, ion transport, and vesicle transport (Yoshimoto *et al.* 2002; Cyert 2003). In the major pathogenic fungi, including *Candida albicans* and *Aspergillus fumigatus*, the calcineurin signaling pathway plays crucial roles in virulence and stress responses (Bader *et al.* 2003; Blankenship *et al.* 2003; Steinbach *et al.* 2006, 2007a,b; Lee *et al.* 2013). Loss of calcineurin causes increased cell sensitivity to stresses including high temperature, salt, cell wall stress, and also results in attenuated or loss of virulence in human pathogenic fungi. Therefore, calcineurin is considered a target for anti-fungal drug discovery, and, in fact, the immunosuppressants FK506 and CsA show anti-fungal activity (Brizuela *et al.* 1991; Odom *et al.* 1997a). A series of studies also demonstrated that the Crz1 orthologs are prominent calcineurin downstream targets in pathogenic fungi (Onyewu *et al.* 2004; Cramer *et al.* 2008; Schumacher *et al.* 2008; Soriani *et al.* 2008; Miyazaki *et al.* 2010).

*Cryptococcus neoformans* is a ubiquitous fungus that is widespread in the environment, including associations with trees, soil, and bird guano (Monari *et al.* 1999; Idnurm *et al.* 2005; Chang *et al.* 2014). This fungus is a basidiomycetous fungus and both its spores and desiccated yeast cells serve as infectious propagules (Hung and Schreiber 1992; Kwon-Chung *et al.* 2014). *C. neoformans* is an opportunistic pathogenic fungus that causes meningoencephalitis in patients who have weakened immune systems, including HIV/AIDS and organ transplant patients, leading to high mortality (Kidd *et al.* 2004; Park *et al.* 2009). In this human pathogenic fungus, calcineurin is a key virulence factor that is required for adaptation to stressful host environments, including elevated temperature (Odom *et al.* 1997b; Fox *et al.* 2001). In addition, calcineurin is essential for stress adaptation, sexual reproduction, and virulence of *C. neoformans* (Fox *et al.* 2001; Danielsen *et al.* 2013). During thermal and other stress conditions, calcineurin colocalizes with components of P-bodies/stress granules (PBs/SGs) (Kozubowski *et al.* 2011a,b), which consist of aggregates of RNA binding proteins, mRNA decay machinery, and translation initiation factors (Buchan and Parker 2009; Mitchell *et al.* 2013; Buchan 2014).

Our recent phosphoproteomic analysis identified 44 putative calcineurin targets in *C. neoformans* (Park *et al.* 2016). We demonstrated that the Crz1 ortholog is a *bona fide* calcineurin target in *C. neoformans*. Under thermal stress conditions, calcineurin controls the transcriptional activity and nuclear translocation of Crz1 through dephosphorylation (Park *et al.* 2016). In addition, we proposed that several RNA binding proteins, including Pbp1 (PAB1-binding protein 1), Puf4 (PUmilio-homology domain Family 4), and Lhp1, are potential calcineurin targets. Pbp1 is involved in fungal virulence and sexual reproduction (Park *et al.* 2016). Both Puf4 and Lhp1 are required for heat stress survival (Park *et al.* 2016). Employing epistasis analyses, we demonstrated that two downstream branches of the calcineurin pathway govern cell viability at high temperature, sexual reproduction, and fungal virulence (Park *et al.* 2016).

Although we characterized several calcineurin targets, including Crz1 and RNA binding proteins, in our previous study (Park *et al.* 2016), many putative targets await characterization. To further characterize the remaining calcineurin targets revealed by the above study, we examined stress responses of 13 putative calcineurin-target mutants from a systematic deletion mutant library of *C. neoformans* (Liu *et al.* 2008). Among them, the CNAG_01744∆ mutant showed increased sensitivity to cell wall stress in comparison to the WT. Based on BLAST analysis, CNAG_01744 shares considerable homology with the *S. cerevisiae* Had1 protein. Had1 contains a haloacid dehalogenase (HAD) domain, and is a member of the β-phosphoglucomutase family of proteins that is highly conserved in most organisms (Burroughs *et al.* 2006; Kuznetsova *et al.* 2015). In yeast, several β-phosphoglucomutase family proteins, including two 2-deoxyglucose-6-phosphatase (Dog1 and Dog2) and two glycerol-1-phosphate phospohydrolases (Rhr2/Gpp1 and Hor2/Gpp2), are well characterized and are required for cellular responses to environmental stresses such as osmotic and oxidative stresses (Randez-Gil *et al.* 1995a,b; Norbeck *et al.* 1996; Kuznetsova *et al.* 2015). Given the relevance of HAD in stress responses, we were prompted to characterize the role of Had1 in the calcineurin pathway functions in *C. neoformans*. Our results demonstrate that loss of Had1 causes increased sensitivity to thermal or cell wall stresses and attenuated virulence. To dissect the link between Had1 and Crz1 in calcineurin-related functions, we tested the impact of the *had1*∆ *crz1*∆ double mutation. The results show that Crz1 and Had1 play additive roles in thermoresistance and virulence. However, whether Had1 is a direct calcineurin substrate remains to be established.

## Materials and Methods

### Strains, media, and culture conditions

Fungal strains used in this study are listed in Table 1. Liquid and solid yeast extract-peptone-dextrose (YPD; Difco, Sparks, MD) media were used for general cultures of *C. neoformans*. To assay thermo-tolerance, fungal cells grown overnight at 30° were 10-fold diluted, spotted on YPD plates, and then cultured at distinct temperatures (30, 37, 38, and 39°). To examine susceptibility to other stresses, 2.5–5 μl of cultured cells grown in liquid YPD medium overnight were ten-fold serially diluted and spotted on YPD medium containing the indicated concentration of the following compounds; Congo red (CR; Sigma, St. Louis, MO) and sodium dodecyl sulfate (SDS; Fisher, Fair Lawn, NJ) for membrane destabilizing stress; dithiothreitol (DTT, Sigma) for reducing stress; calcofluor white (CFW; Sigma) for chitin synthesis inhibition, which results in cell wall stress, NaCl (Fisher); and KCl (Fisher) for salt stresses. Fungal cells were incubated at 30° and photographed post-treatment from d 2 to d 3.

**Table 1 C.** *neoformans* strains used in this study

Strains	Relevant Genotype	Reference
H99	MATalpha, Wild type	Perfect *et al.* (1980)
HP235	MATalpha *crz1*∆::NEO	Park *et al.* (2016)
HP242	MATalpha *cna1*∆::NEO	Park *et al.* (2016)
HPC23	MATalpha *had1*∆::NEO	This study
HPC24	MATalpha *had1*∆::NEO	This study
HPC27	MATalpha *had1*∆::NEO *HAD1_4xFLAG*::HYG	This study
HPC28	MATalpha *had1*∆::NEO *HAD1_4xFLAG*::HYG	This study
HPC29	MATalpha *crz1*∆::NAT *had1*∆::NEO	This study
HPC30	MATalpha *crz1*∆::NAT *had1*∆::NEO	This study
HPC25	MAT**a** *had1*∆::NAT	This study
HPC26	MAT**a** *had1*∆::NAT	This study
KN99	MAT**a**, Wild type	Nielsen *et al.* (2003)
HP243	MAT**a** *cna1*∆::NEO	Park *et al.* (2016)

### Generation of mutant strains

The oligonucleotides used in this study are listed in Table 2. To generate a deletion mutant, gene deletion cassettes were generated using a double-joint PCR (DJ-PCR) as described (Yu *et al.* 2004). The 5′ and 3′-flanking region of the *HAD1* gene were amplified using primer pairs JOHE42780;JOHE42782 and JOHE42781;JOHE42783, respectively, from the *C. neoformans* serotype A H99 (Perfect *et al.* 1993; Janbon *et al.* 2014) genomic DNA as a template. The selectable markers, *NAT* or *NEO* (Fraser *et al.* 2003), were amplified with the primer pair JOHE40706;JOHE40707 using pAI3 and pJAF1, respectively. The final deletion cassettes were generated by means of DJ-PCR performed using primer pair JOHE42784;JOHE42785 and the 5′ and 3′-flanking regions and markers as templates. The amplified gene deletion cassettes were purified, combined with 0.6 μm gold microcarrier beads (Bio-Rad) using the QIAquick Gel Extraction kit (Qiagen), and then introduced into the wild type (H99 or KN99**a**) mutant strains using biolistic transformation methods (Davidson *et al.* 2002).

**Table 2:** Oligonucleotides used in this study

Name	Sequence (5′–3′)*^a^*	Purpose
JOHE40706	GTAAAACGACGGCCAG	*NAT*, *NEO*, *HYG* markers (M13F)
JOHE40707	CAGGAAACAGCTATGAC	*NAT*, *NEO*, *HYG* markers (M13R)
JOHE42780	GCAGGTGAGCAGTTGTGGCAAG	*HAD1* disruption (5F)
JOHE42781	GACATCCAAATCCCACAATGCC	*HAD1* disruption (3R)
JOHE42782	TCACTGGCCGTCGTTTTAC TTTGGATAATTACTTGGGGGTCTATG	*HAD1* disruption with marker (5R)
JOHE42783	CATGGTCATAGCTGTTTCCTG AATGTGTTAAATGTAGCGATAGGC	*HAD1* disruption with marker (3F)
JOHE42784	GACGGCTCGTTACTGTGTTAGATTG	*HAD1* disruption (NF_Nested)
JOHE42785	CAAGATCCCAGTGTCGTGGAG	*HAD1* disruption (NR_Nested)
JOHE42786	aatt **GCGGCCGC** CTATTGTGAGCTACTGGCCTGGTG	5′ *HAD1* with promoter and *Not*I
JOHE42787	aatt **GCGGCCGC** CTCGTCCTGAGACATTTCGCCTTG	3′ *HAD1* with *Not*I
JOHE41391	GGTACTCACAACTGAGCCAGCAG	*CRZ1* disruption (5F)
JOHE41392	TCATCGTCGTCGAAGTCGAGGC	*CRZ1* disruption (3R)
JOHE41393	TCACTGGCCGTCGTTTTAC GTGGATTATAGGGGTGACTGATAGA	*CRZ1* disruption with marker (5R)
JOHE41394	CATGGTCATAGCTGTTTCCTG CGATGGTCATAGGGCGCTGTGAG	*CRZ1* disruption with marker (3F)
JOHE41395	GGTTCGTTAGTCGGGTCAACTG	*CRZ1* disruption (NF)
JOHE41396	TTAGGGGAGGTTGGGATCGG	*CRZ1* disruption (NR)

a Underlined sequence is homologous to vector or cassette sequence, lowercase indicates linker sequence, bold sequence denotes restriction site sequence.

To generate the double deletion mutants, 5′ and 3′-flanking regions for *CRZ1* (JOHE41391;JOHE41393 and JOHE41392;JOHE41394) were amplified. The *NAT* marker was used for the disruption cassettes. After fusion by DJ-PCR, *crz1* disruption constructs were amplified using JOHE41395;JOHE41396, and introduced into HPC24 (*had1*∆ mutant). Multiple stable transformants were isolated from independent experiments, and were selected on YPD medium containing nourseothricin sulfate or G418, and then confirmed by diagnostic PCR for the 5′ and 3′ junctions, followed by restriction enzyme digestion.

To express the Had1-FLAG fusion protein, the *HAD1* gene region, including its predicted promoter but lacking its termination codon, was amplified using the primers JOHE42786 and JOHE42787. The PCR product was then digested with *Not*I and cloned into pHP2 (Park *et al.* 2016), which contains a 4× FLAG tag, the *HOG1* terminator, and the hygromycin B-resistance gene. The resulting plasmid pHSP1 was then introduced into the recipient *had1*∆ strains. Multiple transformants were selected on YPD medium containing hygromycin B (Sigma), and then confirmed by PCR and Western blot analyses.

### Had1 mobility assay

Had1 mobility assay was conducted as described previously (Park *et al.* 2016). Strains expressing Had1-FLAG were grown in YPD at 25° to an optical density OD_600_ 0.6–0.8, and cultures were grown at 25° or shifted from 25 to 37° for 1 hr with or without FK506 (2 μg/ml). Cells were collected and disrupted in lysis buffer (50 mM Tris-HCl, pH 7.5, 150 mM NaCl, 0.5 mM EDTA, 0.5% Triton X-100) supplemented with a protease inhibitor tablet (Roche) and phosphatase inhibitor cocktails (Thermo) using a bead beater for 10 cycles (60 sec homogenization with 60 sec rest). Cell lysates were centrifuged for 15 min at 14,000 × *g*, the supernatant was recovered, and protein concentration was determined by employing the Bio-Rad Bradford reagent. The supernatant was subjected to SDS-PAGE and transferred to PVDF membranes (Bio-Rad). For western blot analysis, we employed mouse monoclonal anti-FLAG M2 antibodies (Sigma), anti-mouse antibody conjugated to horseradish peroxidase (Thermo), and ECL western blotting detection reagent (GE healthcare).

### Virulence assay

*Cryptococcus* strains were cultured overnight in liquid YPD medium at 30°. The resulting fungal cells were collected, washed with sterile PBS, counted with a hemocytometer, and the final density was adjusted to 1 × 10^7^ colony forming units (CFU)/ml. Six- to 7-wk-old Female BALB/c mice were purchased from the Daehan BioLink Co., Ltd., Korea, and used for infection and fungal burden assays. Intranasal infection was performed as previously described (Cox *et al.* 2000). Fourteen mice were anesthetized, and infected intranasally with 5 × 10^5^ CFU in a volume of 50 μl as previously described (Cox *et al.* 2000).

For survival tests, groups of 10 mice were used. Survival was monitored daily, and moribund mice were killed with CO_2_. Survival curves were generated using the Kaplan-Meier method using Prism 4.0 (GraphPad software), and statistical significance (*p* values) were assessed with the log-rank test.

For fungal burden assays, infected mice (three to four) were killed by exposure to CO_2_. Lung and brain tissues were isolated, placed in saline, and then homogenized. The suspension was serially diluted with saline, plated onto YPD agar supplemented with antibiotics (kanamycin, ampicillin, and spectromycin), and incubated at 30° for 3 d. Colony counts were performed and adjusted to reflect the total lung or brain CFU. Statistical analysis was performed using the Student’s *t*-test to assess statistically significant differences between the samples,.

### Ethics statement

Animal care and all experiments were conducted in accordance with the ethical guidelines of the Ethics Review Committee for Animal Experimentation (ERCAE) of Handong Global University (HGU). The HGU ERCAE approved the entire vertebrate animal protocol (protocol #HGU-20160616-009).

### Data availability

All the strains and plasmids used in this study are available upon request. The authors state that all data necessary for confirming the conclusions presented in the article are represented fully within the article.

## Results

### CNAG_01744 encodes a HAD-like hydrolase protein (Had1)

**Figure 1:**
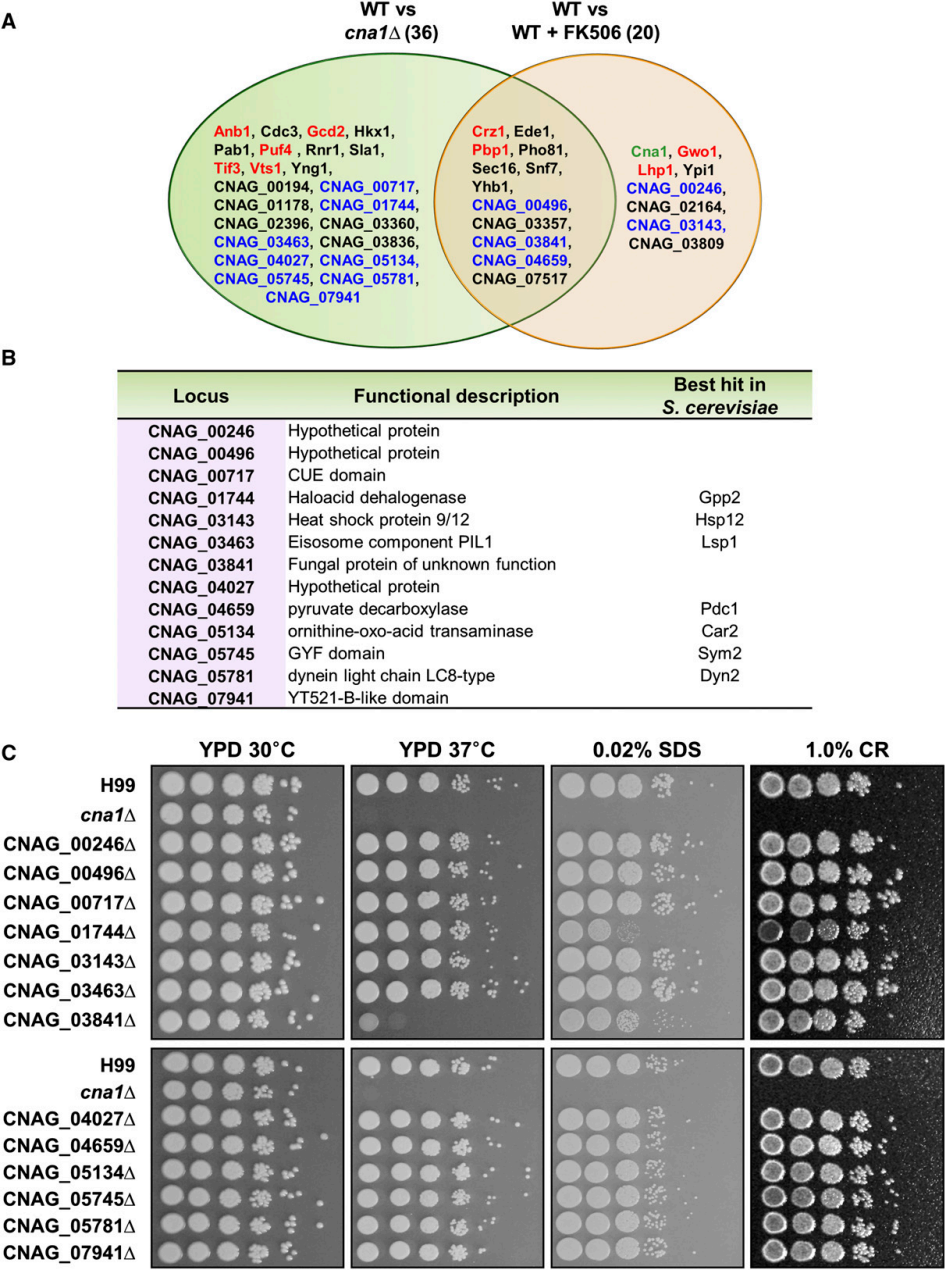
Phenotypes of the potential calcineurin target mutants exposed to various stresses. (A) Targets in the Venn diagram labeled red were characterized in the previous study (Park *et al.* 2016). Targets labeled blue were either deleted in our laboratory or obtained from a *C. neoformans* deletion mutant library (Liu *et al.* 2008) and tested in this study. Cna1 (green) is the calcineurin A catalytic subunit. (B) Functional categories ascribed to the potential calcineurin targets tested in this study. (C) Spot dilution assays with WT (H99), *cna1*Δ (HP242), and 13 mutants from a systematic gene deletion library were performed under several stress conditions as indicated. Strain cultures were incubated overnight, serially diluted 10-fold, and plated on YPD medium without or with CR and SDS. Cells were incubated for 2–3 d at 30, or 37° as indicated, and all cultures containing stressor compounds (SDS and CR) were incubated at 30°.

Previously, we performed phosphoproteomic analyses and identified 44 putative calcineurin targets. Among these targets, we generated deletion mutants for eight, and characterized these as authentic calcineurin targets (Park *et al.* 2016). In the present study, 13 additional putative calcineurin targets identified by the above phosphoproteomic analysis mutants were available in a systematic deletion mutant library of *C. neoformans* (Liu *et al.* 2008) (Figure 1, A and B), and these were selected and analyzed for calcineurin-related phenotypes. Because calcineurin is essential for responses to stress, these mutants were subjected to phenotypic analyses under several stress conditions. The CNAG_01744∆ and CNAG_03841∆ mutants exhibited increased sensitivity to cell wall stress (SDS and CR) as compared to the wild type (WT) (Figure 1C). CNAG_03841 encodes a hypothetical protein and CNAG_01744 encodes a HAD-like hydrolase protein. In this study, we focused on characterizing the functions of CNAG_01744.

The protein sequence of the CNAG_01744 open reading frame (ORF) was blasted against the *S. cerevisiae* S288 genome database and was found to bear identity to six HAD β-phosphoglucomutase family proteins (Supplemental Material, Figure S1A). Furthermore, *C. neoformans* genome blast searches revealed a total of four ORFs that feature HAD domains, including CNAG_01744, CNAG_06122, CNAG_06132, and CNAG_06698 (Figure S1B). CNAG_01744 showed the highest homology (Score 110, identity 32%) to *S. cerevisiae* Gpp2. However, we found that CNAG_06122 shows much higher homology (Score 119, identity 33%) to *S. cerevisiae* Gpp2 (a glycerol-1-phosphatase induced in response to osmotic and oxidative stress) than CNAG_01744. Therefore, CNAG_01744 was named Had1 instead of Gpp2.

### Had1 is essential for appropriate stress response and may function independently of calcineurin

**Figure 2:**
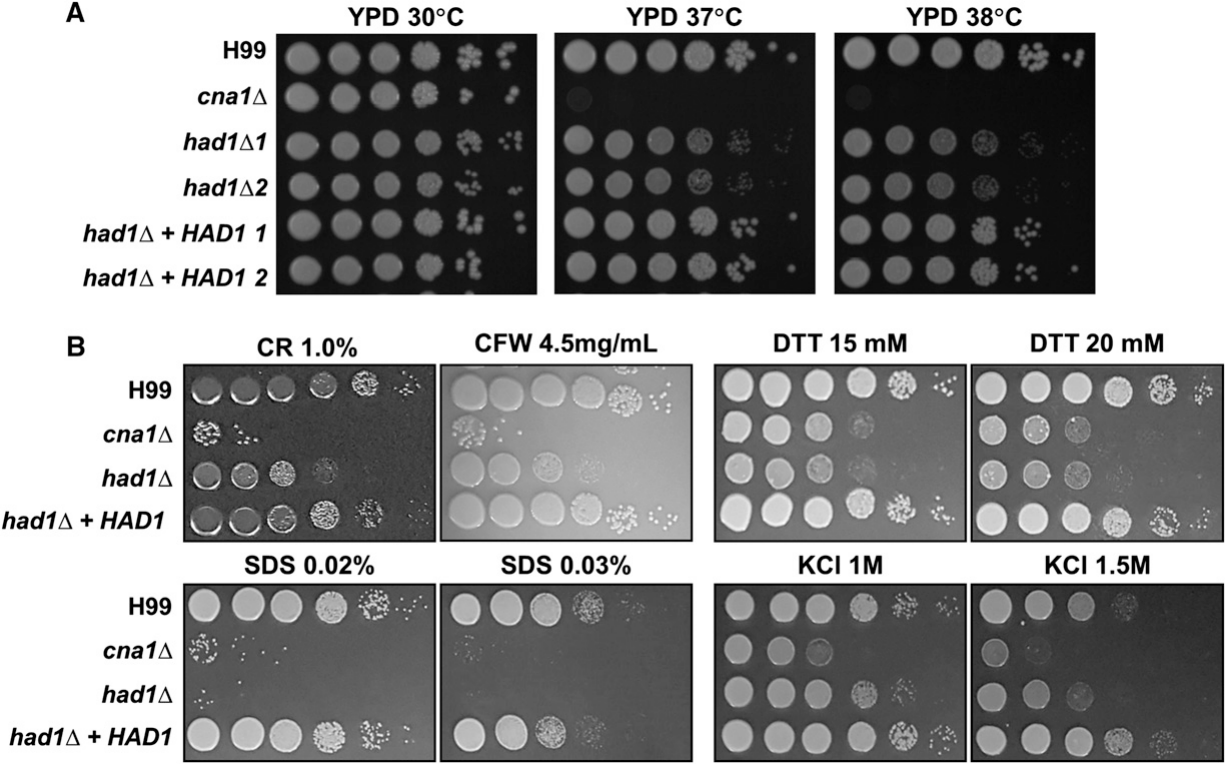
Deletion of *had1* results in hypersensitivity to various stresses. Spot dilution assays with WT (H99), *cna1*Δ (HP242), and *had1*Δ mutants (HPC23 and HPC24), and *had1*∆ + *HAD1* complemented strains (HPC27 and HPC28), were performed under several stress conditions as indicated. (A) Cell cultures in liquid YPD medium were grown overnight at 30° and serially diluted 10-fold. Equal aliquots were spotted on solid YPD medium supplemented, or not, with the stressor compounds as indicated in (B). (A) For thermotolerance, cells were incubated on solid medium for 2 d at 30, 37, or 38° as indicated. (B) For cell wall, endoplasmic reticulum, or osmolarity stress, the solid YPD culture medium was supplemented with CR, CFW, DTT, SDS, or KCl at the indicated concentrations, and the cultures were incubated at 30°. Results shown are representative of two independent experimental replicates.

To test the functions of Had1, we generated a *had1* deletion (*had1*∆) mutant, and complemented strains, and examined their phenotypes under a variety of temperature conditions. As mentioned above, we confirmed that the *had1*∆ mutant exhibited increased sensitivity to high temperatures when compared to the WT strain (Figure 2A). To further examine the role of Had1, we tested the growth phenotypes of *had1* mutants on solid media containing various cell stressors agents including CR, CFW, SDS, DTT, and KCl. As shown Figure 2B, the *had1*∆ mutant showed increased sensitivity to cell wall perturbing agents, in particular to SDS, as compared to the WT strain, suggesting that Had1 may be involved in controlling stress response.

Because Had1 is a potential calcineurin target identified from the phosphoproteomics results (Park *et al.* 2016), we examined the migration of Had1 under both heat or high salt osmotic stress conditions. Cultures of yeast cells expressing FLAG tagged Had1 were cultured at 25° and shifted (or not) either to 37° for 1 hr, or to medium containing 1 M NaCl, and incubated at 25° for 1 hr. For both stress conditions, cultures were treated (or not) with FK506 for 1 hr. Interestingly, The Had1-FLAG protein isolated from cells shifted from 25 to 37° or from cells exposed to high salt osmotic stress, displayed reduced gel migration as compared to that isolated from cultures grown at 25° (Figure S2A). However, FK506 did not affect the gel migration of Had1 under either stress condition (Figure S2). These results suggest that the stress-induced post-translational modification resulting in altered Had1 gel mobility is independent of calcineurin, but do not rule out the possibility that Had1 is a substrate of calcineurin that cannot be detected by gel mobility shift assay.

### Had1 and Crz1 play an additive role in stress response and virulence

**Figure 3:**
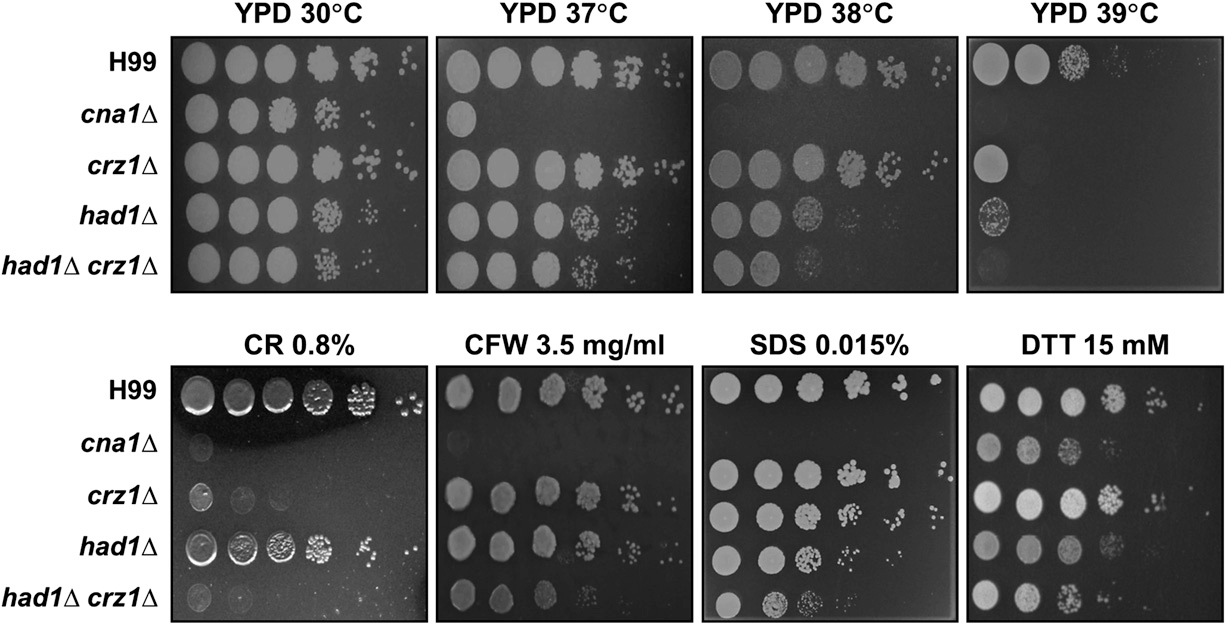
Had1 and Crz1 orchestrate stress response. Stress tolerances of WT (H99), the single *cna1*Δ (HP242), *crz1*Δ (HP235), and *had1*Δ (HPC24) mutants, or double *had1*∆ *crz1*Δ (HPC29) deletion mutant strains. Cells were grown overnight at 30°, serially diluted 10-fold, and plated on YPD medium. Plates were incubated for 2 d at 30, 37, 38, or 39° as indicated.

Previously, our study demonstrated that calcineurin regulates high temperature growth and virulence via transcriptional and post-transcriptional processes (Odom *et al.* 1997b; Cruz *et al.* 2001). Among the characterized calcineurin targets, Crz1 is the only known target involved in cell wall integrity. To examine whether Crz1 and Had1 function in a single pathway or in parallel pathways within the signaling cascades governing cell wall integrity, we generated *had1 crz1* double deletion mutants, and examined their phenotypes during stress responses. As shown Figure 3, the *had1*∆ *crz1*∆ double mutant strains exhibited increased sensitivity to various stresses compared to either the *crz*∆*1* or *had1*∆ single deletion mutant. These results demonstrate that Had1 and Crz1 play an additive role in thermotolerance and cell wall stresses, and suggest they operate in parallel pathways.

### Had1 is involved in fungal pathogenicity

**Figure 4:**
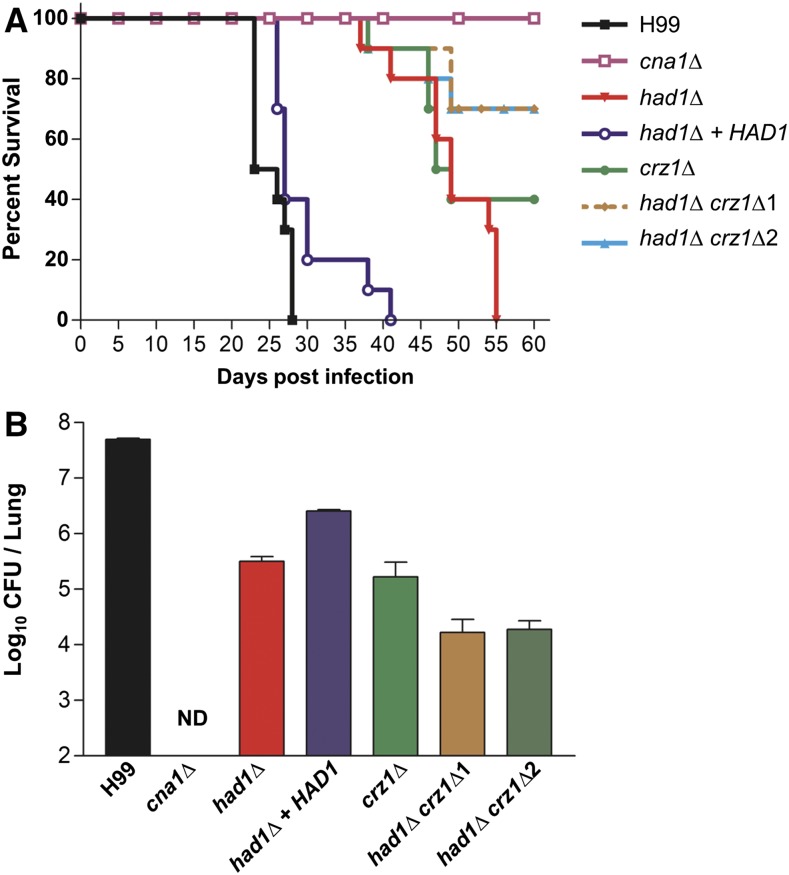
Had1 is required for virulence. (A) Virulence was tested for the WT (H99), *cna1*Δ (HP242), *crz1*Δ (HP235), *had1*Δ (HPC24), *had1*∆ + *HAD1* (HPC28), and *had1*∆ *crz1*Δ (HPC29 and HPC30) strains. Yeasts cells were grown overnight in YPD liquid medium at 30°, collected, washed with PBS, and 5 × 10^5^ cells were inoculated into female BALB/c mice via intranasal instillation. Animal survival was monitored for 60 d postinfection. (B) Mice (*n* = 3∼4) were inoculated intranasally with 5 × 10^5^ CFU of the indicated strains. Lungs were isolated at 14 d postinfection. Results are expressed as mean CFU per lung ± SEM. ND, not detected.

Because of hyper-susceptibility to heat and other stress conditions, we hypothesized that Had1 would be required for pathogenicity of *C. neoformans*. To examine virulence of these mutants, we conducted animal virulence studies using a murine inhalation model. As shown in Figure 4A, the *had1*∆ mutant exhibited attenuated virulence compared to the WT strains. Virulence was restored to nearly WT levels when the *had1*∆ mutant was complemented by reintroduction of the WT *HAD1* gene in the *had1*∆ + *HAD1* strain. The *had1*∆ *crz1*∆ double mutant strains were more attenuated in virulence as compared with either the *had1*∆ or the *crz1*∆ single mutant (Figure 4A). These results demonstrate that Had1 contributes significantly to *C. neoformans* virulence.

To further examine the virulence defect conferred by the *had1*∆ mutation, fungal burden in the lungs and brain of mice infected with WT and *had1*∆ mutant strains was analyzed at d 14 post infection (Figure 4B). The *cna1*∆ mutant exhibited an undetectable fungal burden in both lung and brain tissues. By comparison, the *had1*∆ and *crz1*∆ single mutant strains showed reduced fungal burden in the lung compared with the WT strain. Moreover, the *had1*∆ *crz1*∆ double mutant strains exhibited significantly reduced fungal burden in the lung compared with either single deletion mutant strain. While we recovered WT and mutant strains from the lung, we did not recover any yeast cells from brain tissue 14 d after infection for both WT and mutant strains (data not shown). Collectively, these findings demonstrate that Had1 is required for full virulence, and that Had1 and Crz1 function in parallel pathways controlling virulence.

## Discussion

**Figure 5:**
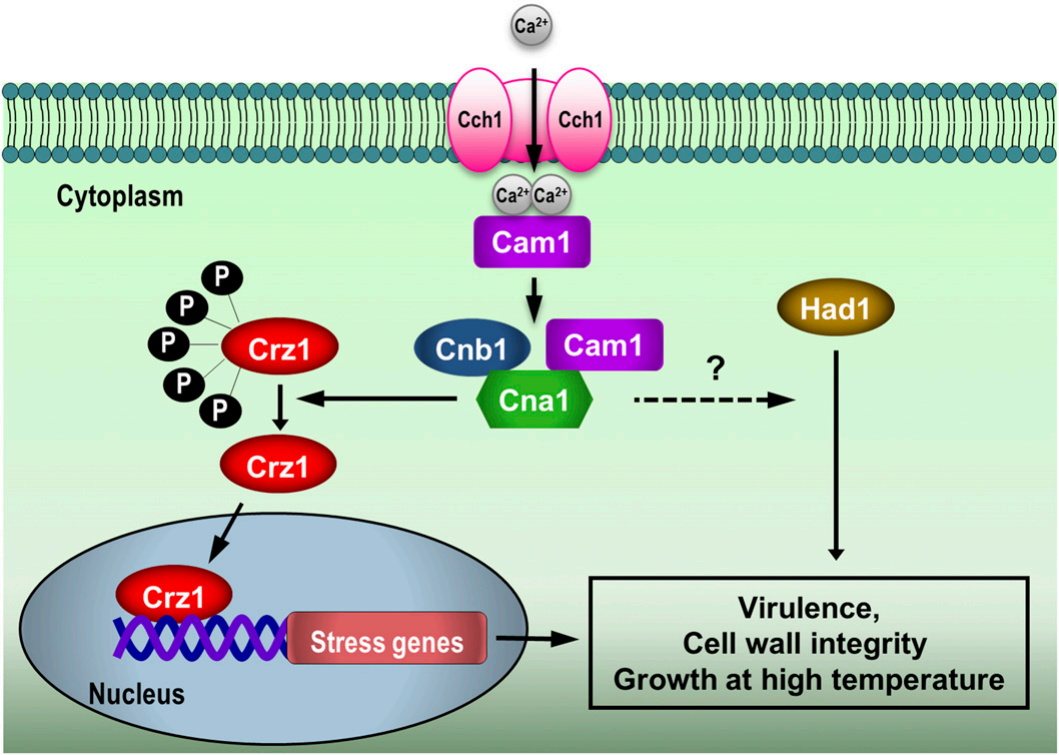
The calcineurin-Crz1 pathway and Had1 control growth at high temperature, stress response, and virulence via parallel pathways. The calcineurin signaling network acts via Crz1 to control *Cryptococcus* thermotolerance, cell-wall integrity, and virulence. Had1 also contributes to these functions in a pathway parallel to Crz1, and possibly regulated by calcineurin. Dotted line arrow and question mark denote a pathway step as yet not well defined, as it remains to be determined whether Had1 is an authentic calcineurin target.

Given that calcineurin is a key pathway regulating stress responses, mating, and fungal virulence, identification and characterization of calcineurin targets is crucial for understanding the calcineurin signaling network (Aramburu *et al.* 2004; Steinbach *et al.* 2006; Goldman *et al.* 2014). In a previous study, we identified Had1 as a potential target of calcineurin in *C. neoformans* (Park *et al.* 2016). In the present study, although we were unable to confirm that Had1 is a calcineurin target, *had1* mutation resulted in hypersensitivity to thermal and other stresses, and attenuated virulence, suggesting that Had1 is important for stress responses and virulence. Moreover, double deletion mutant analysis suggests that Had1 and Crz1 function in parallel pathways controlling fungal pathogenicity and cell wall stress responses (Figure 5). However, we could not determine if Had1 functions dependently or independently of calcineurin. The preliminary results of our Had1 gel mobility analysis showed that, while both thermal and osmolarity stresses seem to affect Had1 mobility, in both cases, this is not altered by FK506, and, thus, it seems to be independent of calcineurin (Figure S2). Our previous phosphoproteomic screen revealed only one calcineurin-dependent phosphorylation site in Had1 (Park *et al.* 2016). Therefore, it is likely that this single site could not be detected by our mobility shift assay (Figure S2), which is more suited to detecting multiple phosphorylation sites on a protein. Thus, the present study does not rule out the possibility that Had1 function is controlled by calcineurin; testing this model will require more sensitive assays and further analysis.

HAD-like hydrolases are large superfamilies of enzymes that exhibit phosphatase, ATPase, phosphonatase, and phosphomutase activity, and are conserved in both prokaryotic and eukaryotic organisms (Burroughs *et al.* 2006; Kuznetsova *et al.* 2015). Forty-five genes encoding HAD-like hydrolases are found in the yeast genome, and 15 of their protein products have been biochemically characterized for this enzymatic activity (Randez-Gil *et al.* 1995a,b; Norbeck *et al.* 1996; Kuznetsova *et al.* 2015). Among these, the β-phosphoglucomutase family proteins, such as Dog1, Dog2, Gpp1, and Gpp2, are well characterized in yeast. These proteins are required for cellular responses to environmental stresses. The expression of Gpp1, Gpp2, and Dog2 is induced by environmental stresses, including osmotic or oxidative stresses, and by glucose starvation (Tsujimoto *et al.* 2000; Pahlman *et al.* 2001). Interestingly, the expression of these genes is controlled by either the HOG (High Osmolality Glycerol) or the Snf1 kinase pathways (Tsujimoto *et al.* 2000; Pahlman *et al.* 2001). In addition, loss of both the *GPP1* and *GPP2* genes results in hyper-sensitivity to oxidative stress (Pahlman *et al.* 2001). However, *gpp1*Δ, *gpp2*Δ, and *dog2*Δ single mutants did not show any increased susceptibility to osmotic or oxidative stresses. Our study establishes that Had1 is required for proper responses to heat and osmotic stresses, and this finding is in accord with previous studies suggesting this function. Previous microarray analyses shown that the expression of *HAD1* (formerly *GPP1*) was induced in response to osmotic stress, and this microarray data were confirmed by our qRT-PCR analysis (Figure S3A). Ko *et al.* (2009) also demonstrated that induction of *HAD1* in response to osmotic stress was decreased in *hog1* and *ssk1* mutants, suggesting that the expression of *HAD1* is controlled by the stress-activated Hog1 signaling pathway in *C. neoformans* (Ko *et al.* 2009). These results suggest that the regulatory mechanism of *HAD1* mRNA expression in response to osmotic stress is conserved in *S. cerevisiae* and *C. neoformans*. We demonstrate here that, upon thermal stress, the expression of *HAD1* slightly increases (Figure S3B). To examine whether the *HAD1* expression is regulated by the calcineurin-Crz1 pathway, we searched our published transcriptome analysis (Chow *et al.* 2017), and found that *HAD1* expression is regulated independently of calcineurin or Crz1 under thermal stress.

Previously our phosphoproteomic screen revealed that Had1 is a potential calcineurin substrate in *C. neoformans*. The single calcineurin-dependent phosphorylated peptide (RRA**S**_376_QSGQAGVTLDAFRR) in Had1 was increased more than twofold in abundance in the calcineurin *cna1*Δ mutant compared to WT cells (Park *et al.* 2016). In addition, the Had1 protein contains two predicted calcineurin substrate docking sites PxIxIT motifs (P[^PG][IVLF][^PG]) (Park *et al.* 2016). However, as discussed earlier in this section, whether Had1 is a *bona fide* calcineurin target remains to be characterized. Previous studies identified 13 phosphorylation sites located in the Had1 C-terminal region, and some of these residues were proposed to be phosphorylated by the PKA signaling cascade in *C. neoformans* (Selvan *et al.* 2014; Geddes *et al.* 2016). These results indicate that phosphorylation of Had1 is important for Had1 activation, but the detailed mechanisms await further characterization to better understand Had1 roles in stress responses and environmental adaptation. Based on the transcriptomic (Ko *et al.* 2009; Chow *et al.* 2017) and phosphoproteomic (Selvan *et al.* 2014; Geddes *et al.* 2016; Park *et al.* 2016) studies from our and other laboratories, we propose that the expression of *HAD1* mRNA is induced by the HOG signaling pathway in response to environmental stresses (Ko *et al.* 2009), and, in turn, phosphorylation of the Had1 protein may be regulated by the PKA signaling cascade (Geddes *et al.* 2016), and possibly by the calcineurin pathway (Park *et al.* 2016). Activated Had1 is required for appropriate stress responses; however, the detailed mechanisms controlling Had1 function should be further studied to better understand the roles of the (HAD)-like hydrolase superfamily in stress responses, environmental adaptation, and virulence.

## 

## Supplementary Material

Supplemental material is available online at www.g3journal.org/lookup/suppl/doi:10.1534/g3.117.300444/-/DC1.
