# Supplementary figures and images for: Had1 Is Required for Cell Wall Integrity and Fungal Virulence in Cryptococcus neoformans
Source: G3 (Bethesda). 2017 Dec 12;8(2):643–52. doi: 10.1534/g3.117.300444 (PMC5919746; doi:10.1534/g3.117.300444)

Figure S1

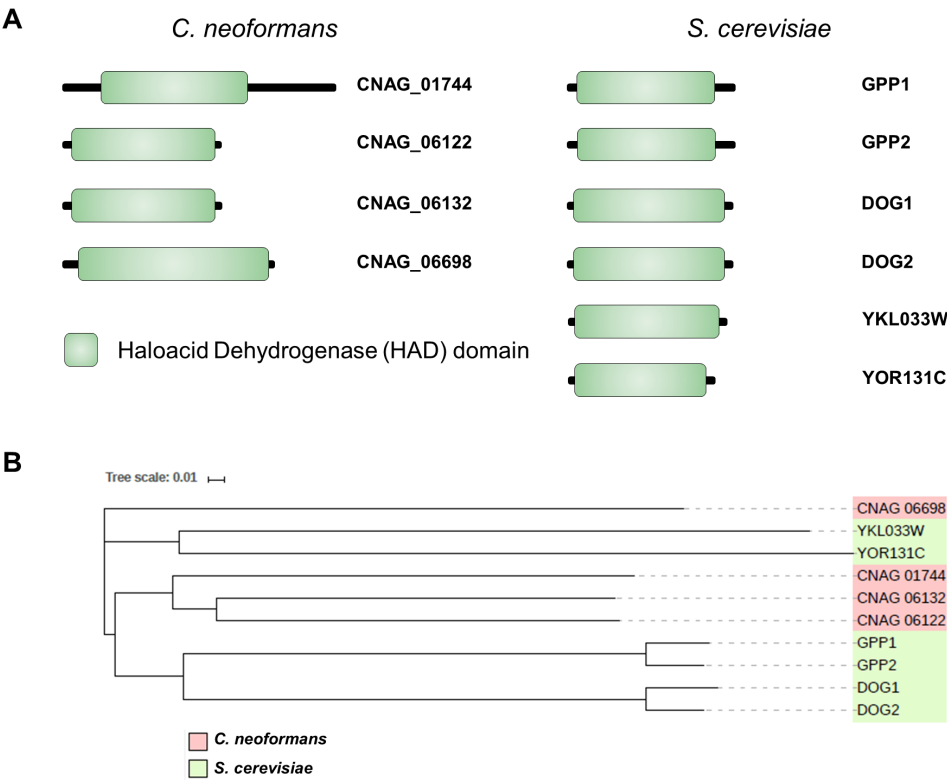

Supplement: Supplementary file 1 [file 643FigureS1.pdf]

Figure S2

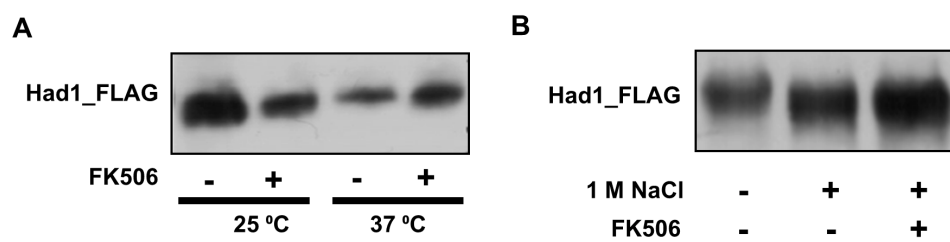

Supplement: Supplementary file 2 [file 643FigureS2.pdf]

Figure S3

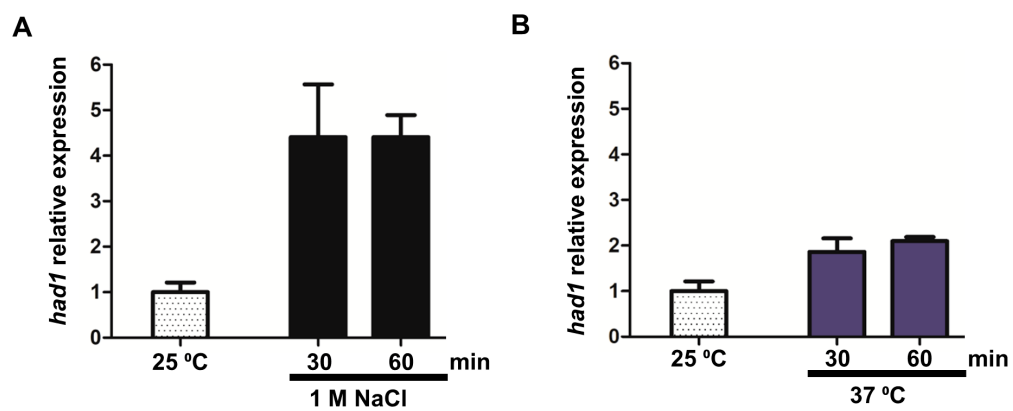

Supplement: Supplementary file 3 [file 643FigureS3.pdf]

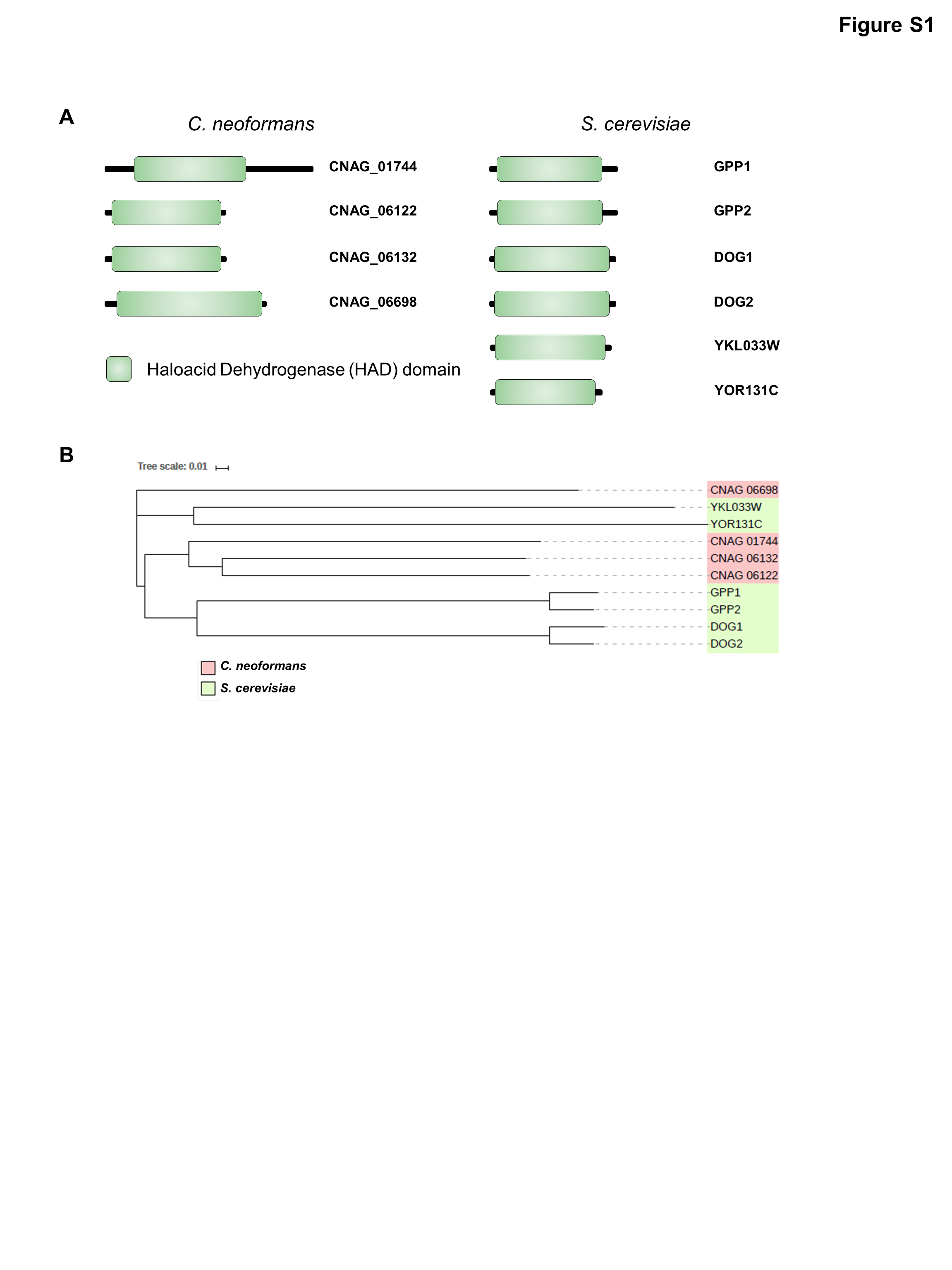

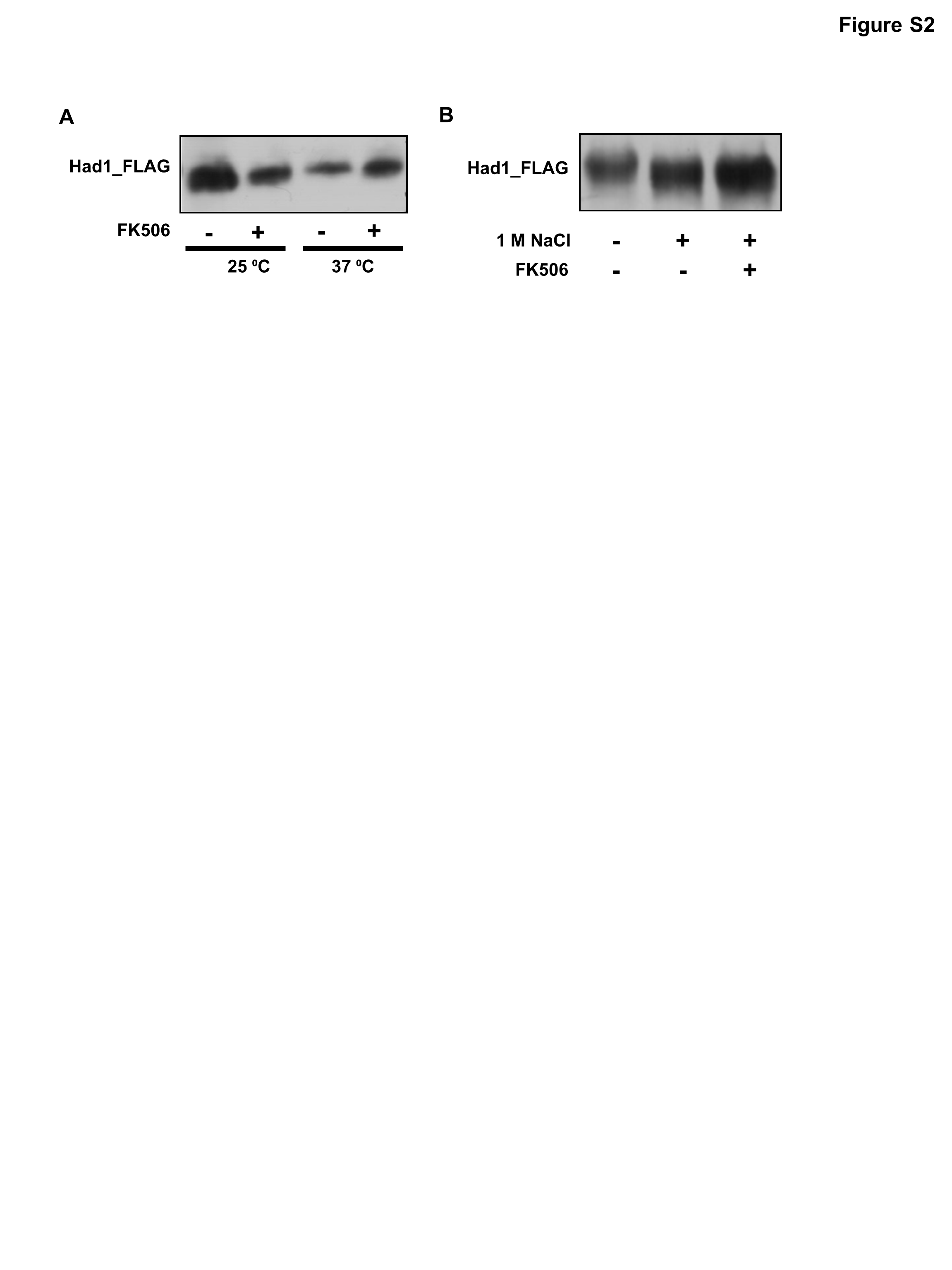

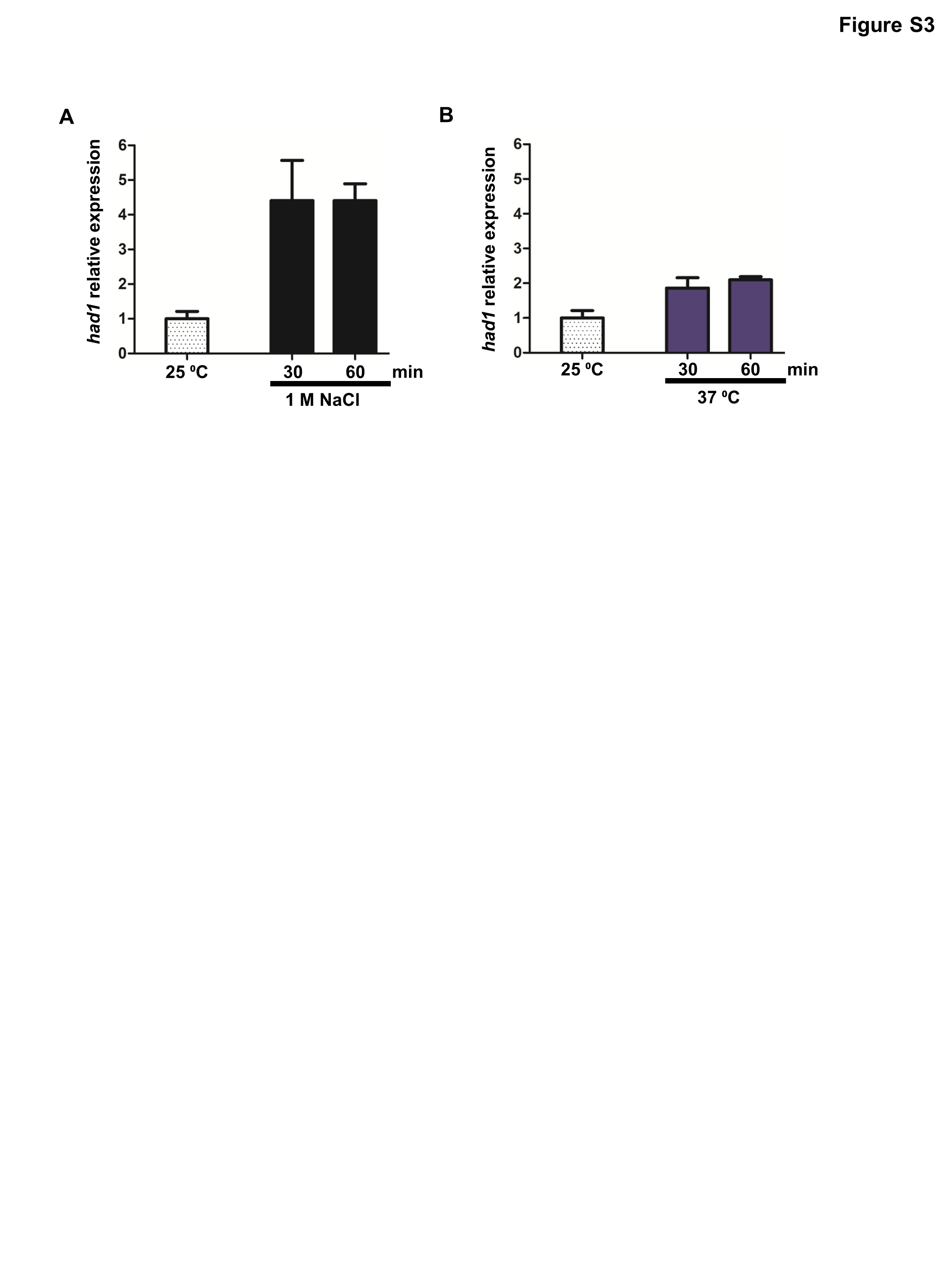

Supplement: Supplementary file 4 [file 643FileS1.docx]
